# Effect of a continuous care model-based program on fatigue, self-efficacy, and quality of life in patients with Parkinson’s disease: study protocol for a randomized controlled trial

**DOI:** 10.1186/s13063-025-09057-5

**Published:** 2025-09-25

**Authors:** Mahshid Ebrahimiyan Tadi, Shahla Abolhassani, Fatemeh Nazari, Ahmad Chitsaz

**Affiliations:** 1 Nursing and Midwifery Care Research Center, Isfahan University of Medical Sciences, Isfahan, Iran; 2 Nursing and Midwifery Care Research Center, Medical Surgical Nursing Department, School of Nursing and Midwifery, Isfahan University of Medical Sciences, Isfahan, Iran; 3 School of Medicine, Department of Neurology, School of Medicine, Isfahan University of Medical Sciences, Isfahan, Iran

**Keywords:** Continuous care, Fatigue, Self-efficacy, Quality of life, Parkinson’s disease

## Abstract

**Background:**

Neurological disorders, including Parkinson’s disease (PD), are among the leading causes of disability worldwide. One of the major challenges of this disease is chronic fatigue, which is considered one of the most common and debilitating non-motor symptoms. Increased fatigue in these patients reduces their self-efficacy, which directly correlates with decreased quality of life (QOL) and worsening motor and non-motor symptoms. Given the profound impact of PD on patients’ daily lives, adopting effective approaches to improve their QOL is essential.

The continuous care model (CCM), designed as an indigenous approach based on Iran’s cultural and social characteristics, can serve as an effective strategy for managing complications associated with PD.

**Methods:**

This study is a clinical trial aimed at evaluating the effect of the CCM on fatigue, self-efficacy, and QOL in patients with PD in Isfahan, Iran. A total of 80 patients will be selected through convenience sampling and randomly assigned to either the intervention or control group. Data will be collected using standardized questionnaires at three time points: before the intervention, immediately after, and 1 month later. In the intervention group, the CCM will be implemented over a nine-week period in four stages: familiarization, sensitization, control, and evaluation. The control group will receive only routine pharmacological care. Data will be analyzed using descriptive statistics (mean and standard deviation), followed by repeated measures ANCOVA to compare changes in outcomes across three time points between groups, controlling for covariates. Normality and homogeneity of variances will be assessed using the Kolmogorov–Smirnov and Levene’s tests, respectively.

**Discussion:**

Various approaches have been proposed to manage fatigue, enhance self-efficacy, and improve QOL in patients with PD; however, many of these strategies face challenges, including the lack of a comprehensive approach. The CCM focuses on the care of patients with chronic conditions, emphasizing education and empowerment for both patients and their families. By placing the patient at the center of care, this holistic approach plays a crucial role in the management of chronic diseases.

**Trial registration.:**

It is registered in the Iranian Registry of Clinical Trials (IRCT) with the registration number IRCT20190712044181N7.

**Supplementary Information:**

The online version contains supplementary material available at 10.1186/s13063-025-09057-5.

## Administrative information

Note: the numbers in curly brackets in this protocol refer to SPIRIT checklist item numbers. The order of the items has been modified to group similar items (see http://www.equator-network.org/reporting-guidelines/spirit-2013-statement-defining-standard-protocol-items-for-clinical-trials/).

**Table:** 

Title {1}	Effect of a continuous care model-based program on fatigue, self-efficacy, and quality of life in patients with Parkinson’s disease: study protocol for a randomized controlled trial.
Trial registration {2a and 2b}.	It is registered in the Iranian Registry of Clinical Trials (IRCT) with the registration number IRCT20190712044181N7.
Protocol version {3}	Protocol, February 6, 2025
Funding {4}	Nursing and Midwifery Care Research Center, Isfahan University of Medical Sciences, Isfahan, Iran
Author details {5a}	Mahshid Ebrahimiyan Tadi^1^, Shahla Abolhassani^2*^, Fatemeh Nazari^3^, Ahmad Chitsaz^4^ ^1^ Nursing and Midwifery Care Research Center, Isfahan University of Medical Sciences, Isfahan, Iran ^2^ Nursing and Midwifery Care Research Center, Adult Health Department, School of Nursing and Midwifery, Isfahan University of Medical Sciences, Isfahan, Iran ^3^ Nursing and Midwifery Care Research Center, Adult Health Department, School of Nursing and Midwifery, Isfahan University of Medical Sciences, Isfahan, Iran ^4^ Research Center of the School of Medicine, Department of Neurology, School of Medicine, Isfahan University of Medical Sciences, Isfahan, Iran ^* **Correspondence to**^ Shahla Abolhassani, Nursing and Midwifery Care Research Center, Adult Health Department, School of Nursing and Midwifery, Isfahan University of Medical Sciences, Isfahan, Iran
Name and contact information for the trial sponsor {5b}	Nursing and Midwifery Care Research Center, Isfahan University of Medical Sciences, Isfahan, Iran Address: Hezar Jerib Street, School of Nursing and Midwifery, Research Centers Complex, Isfahan University of Medical Sciences, Isfahan, Iran, 8,174,673,461 Phone: (+ 98) 31–37,927,621-2/(+ 98) 31–36,699,398 Email: nmrc@nm.mui.ac.ir
Role of sponsor {5c}	This study was funded by the Nursing and Midwifery Care Research Center, Isfahan University of Medical Sciences, Isfahan, Iran. The sponsor had no role in the design of the study, data collection, data analysis, interpretation of the results, manuscript preparation, or the decision to submit the manuscript for publication. All financial and logistical aspects were planned independently by the research team to ensure transparency and full coverage of study-related costs.

## Introduction

### Background and rationale {6a}

PD is the second most common neurodegenerative disorder, with its burden increasing as the global population ages [1, 2]. Over the past 25 years, the prevalence of PD has doubled worldwide, with more than 8.5 million cases recorded in 2019. Additionally, PD has resulted in 5.8 million disability-adjusted life years (DALYs) and 329,000 deaths, showing a significant increase compared to the year 2000 [3]. In Iran, a 2021 study reported that the prevalence of PD among individuals over 50 years old was 261.1 per 100,000, with the highest rate observed in Isfahan [4]. It is estimated that by 2050, the global burden of PD will exceed 12 million cases [5].

PD affects physical, psychological, and cognitive functions, significantly impacting patients, their families, and society [6]. Bradykinesia, muscle rigidity, and postural instability make daily activities challenging, while anxiety, depression, and cognitive issues further reduce QOL and personal independence [7].

In recent decades, the non-motor symptoms of PD, particularly fatigue, have gained significant importance in its diagnosis and management [8]. Fatigue is one of the most common and disabling symptoms of PD, often appearing in the early stages and persisting throughout the disease [9]. Affecting more than half of patients, fatigue leads to weakness, physical deterioration, and reduced ability to work, engage in social activities, and exercise [10, 11]. The prevalence of fatigue among PD patients ranges between 33 and 58% [10] and has been reported to negatively impact their professional and social lives [12]. Many patients consider fatigue to be their most distressing symptom, yet effective treatments remain limited [11]. Therefore, recognizing and managing fatigue is crucial for improving patients’ QOL [10].

Studies indicate that fatigue and other non-motor symptoms of PD are strongly associated with reduced self-efficacy [13]. Self-efficacy, defined as an individual’s perceived ability to perform tasks [13], is generally low in PD patients [14] and plays a key role in disease management and QOL [13]. Among non-motor symptoms, depression and apathy have a greater impact on self-efficacy than motor symptoms and can contribute to its decline even in the early stages of the disease. Patients with lower self-efficacy typically experience a poorer QOL [13].

Poor fatigue management and reduced self-efficacy in PD patients contribute to a decline in QOL [15]. According to the World Health Organization (WHO), QOL encompasses physical health, psychological well-being, social relationships, and environmental factors [16]. Compared to healthy individuals, PD patients experience lower QOL, particularly in terms of physical functioning and mental health [17]. As the disease progresses, it leads to disability, physical and psychological complications, and a further decline in QOL [18].

Several factors significantly impact health-related quality of life (HRQOL) in PD, including depression, disease severity, motor impairments, cognitive dysfunction, and non-motor symptoms such as fatigue and pain. These symptoms worsen as the disease advances, further compromising patients’ well-being [19, 20]. Therefore, implementing effective interventions to enhance the QOL of PD patients is crucial [17].

Current treatments for PD primarily focus on motor symptoms, but a more comprehensive approach is necessary [13]. Integrating nursing care with pharmacological treatment has been shown to reduce depression, improve sleep quality, and enhance patients’ QOL [18]. Continuity of care is a fundamental concept in nursing, referring to the provision of ongoing care to address patients’ health needs [21]. The indigenous model of continuous care, developed by Ahmadi et al., has been effective in improving outcomes such as hospitalization rates, physician visits, lipid levels, and QOL in cardiac patients [22, 23]. This model strengthens the connection between patients and healthcare providers, thereby promoting adherence to healthy behaviors [24].

The CCM consists of four interrelated stages: familiarization (establishing mutual understanding and motivation among patients and their families), sensitization (engaging patients and families with the disease and its complications), control (internalizing healthy behaviors), and evaluation (assessing the model’s effectiveness). This model helps increase patients’ willingness to adopt healthier behaviors [23].

The CCM has been extensively studied in different patient populations, demonstrating positive effects on QOL in heart failure patients [25], self-efficacy in myocardial infarction patients [26], QOL in hemodialysis patients [27], treatment adherence in hemodialysis patients [28], caregiver burden in stroke patients [29], insomnia in pregnant women [30], and sleep quality in older adults [31]. Additionally, a study on multiple sclerosis patients showed that this model significantly improved their QOL [32]. Overall, most studies have reported positive effects of this model on various health outcomes [23].

However, some studies have reported no significant effects of the CCM on certain health outcomes. For instance, one study found that this model did not improve dialysis adequacy in hemodialysis patients [33]. Additionally, research on the QOL of schizophrenic patients indicated that while the model was effective in enhancing interpersonal relationships, it had no significant impact on other dimensions of QOL [34]. Moreover, a study on children with nephrotic syndrome revealed that the model did not reduce the recurrence of the disease [35].

As highlighted earlier, numerous studies have confirmed the positive impact of the CCM on various aspects of disease management, patient outcomes, and caregiver burden. However, some research has reported limited or no effects in specific contexts. Given the high prevalence of PD in Iran and globally, along with the anticipated increase in the aging population, evaluating the effectiveness of this model in reducing disease-related challenges and improving patients’ self-management is crucial. Notably, the CCM, as an indigenous approach tailored to Iran’s cultural and social characteristics, may offer distinct advantages over other methods.

Thus, this study was designed to evaluate the impact of a CCM-based intervention on fatigue, self-efficacy, and QOL in patients with PD.

### Objectives {7}

This study is a randomized controlled clinical trial designed to examine the effectiveness of a CCM-based program on fatigue, self-efficacy, and QOL in patients with PD attending the Parkinson’s Association of Isfahan Province. The effectiveness of this intervention will be assessed using the Parkinson’s Disease Fatigue Scale 16** (**PFS-16), General Self-Efficacy Scale (GSE-17), and Parkinson’s Disease Questionnaire-8 (PDQ-8).

This study aims to improve the condition of patients by implementing a structured intervention based on the CCM. The findings may play a significant role in optimizing care services for individuals with PD.

### Trial design {8}

This study is a randomized controlled clinical trial that includes random allocation of participants, a control group, and an interventional approach to assess the effect of a CCM-based program on fatigue, self-efficacy, and QOL in patients with PD. The study follows a parallel two-group, multivariable design with assessments conducted before the intervention, immediately after, and 1 month post-intervention.

This trial is designed as a superiority trial to evaluate whether the CCM-based program is more effective than standard care in improving fatigue, self-efficacy, and quality of life in patients with Parkinson’s disease.

## Methods: participants, interventions, and outcomes

### Study setting {9}

The Isfahan Parkinson’s Charity Association was selected as the study site due to its accessibility to patients and the presence of documented medical records confirming PD diagnosis.

Established under registration number 6902, this nonprofit organization was founded through the efforts of philanthropists and neurology specialists to support individuals with PD. The association aims to provide education, raise awareness, facilitate prevention, support treatment and rehabilitation, and offer humanitarian assistance to patients and their families.

### Eligibility criteria {10}

#### Inclusion criteria

Patients eligible for participation in the study must meet the following criteria:

- Confirmed diagnosis: diagnosis of PD confirmed by a qualified neurologist specialized in movement disorders, based on the UK PD Society Brain Bank criteria [17].
- Willingness to participate: patients must express willingness to take part in the study [13, 36].
- Cognitive function: absence of cognitive impairment, confirmed by a neurologist’s evaluation [9, 37].
- Physical health: no severe physical illnesses that would prevent participation [38].
- Mental health: no acute or chronic psychiatric disorders [37].
- Neurological health: no history of acute or chronic neurological disorders, such as stroke or traumatic brain injury [37].
- Sensory abilities: no hearing or vision impairments that hinder participation [36].
- Literacy: ability to read and write [39].
- Medication use: no current use of psychiatric medications [39].
- Language: native Persian speakers.
- Family involvement: at least one family member must attend educational sessions alongside the patient.
- No concurrent interventions: patients must not participate in any other educational sessions or treatment-care interventions related to PD during the study period.

#### Exclusion criteria

Patients will be excluded from the study if they meet any of the following conditions:

- Hospitalization: need for hospital admission during the study period.
- Absenteeism: absence from more than two sessions [39].
- Lack of cooperation: failure to actively participate or express unwillingness to continue [39].
- Cognitive decline: development of cognitive impairments during the study [9].

### Who will take informed consent? {26a}

Before participation, all eligible patients will receive both oral and written information about the study, including its objectives, procedures, potential risks and benefits, and their rights as participants. Adequate time will be given for questions and consideration.

Informed written consent will be obtained by the researcher, Ms. Mahshid Ebrahimiyan Tadi, a master's student in nursing and the person responsible for conducting the intervention.

### Additional consent provisions for collection and use of participant data and biological specimens {26b}

Not applicable. No biological specimens will be collected, and no additional consent is required for ancillary studies.

## Interventions

### Explanation for the choice of comparators {6b}

The control group will receive routine care, which represents the standard nursing and medical practices currently implemented in the study setting. This comparator was chosen to evaluate the effectiveness of the intervention against the usual level of care and to ensure the results are applicable to real-world clinical conditions.

### Intervention description {11a}

After obtaining approval from the School of Nursing and Midwifery, Isfahan University of Medical Sciences, and presenting it to the officials of the Parkinson’s Charity Association, the researcher will enter the study setting and select participants based on inclusion criteria and neurologist confirmation. After obtaining written informed consent, participants will complete baseline questionnaires (demographic and clinical information form, PFS-16, GSE-17, and PDQ-8) in a 30–45-min session.

Participants will then be randomly assigned to either the intervention group (*n* = 40) or the control group (*n* = 40) using block randomization. After completing consent forms and baseline assessments, the CCM—comprising four stages: familiarization, sensitization, control, and evaluation—will be implemented for the intervention group over a period of 8 weeks (2 months).

The control group will receive no intervention and will continue with standard medical treatment and routine care as prescribed by their physician [40].

In the first phase of the CCM, known as the familiarization stage, the study participants in the intervention group (including the patient and their caregiver) attend a session lasting approximately 30 to 45 min. This session takes place in a quiet room with appropriate lighting and temperature, free from environmental distractions, within the Parkinson’s Association.

During this stage, the nurse is introduced to the patient and their caregiver, and the expectations of the nurse at different stages of the study are outlined. Additionally, the expectations and needs of the patient and caregiver are identified. Recommendations are provided regarding the importance of continuity in the caregiving relationship and minimizing interruptions until the designated period ends. Moreover, the schedule for in-person and telephone follow-ups is established, and the objectives and means of communication, along with the structure of educational sessions, are explained [40].

The second phase of the CCM, known as the sensitization stage, aims to enhance the awareness of patients and their families regarding the nature of the disease, its early and late complications, preventive measures, and associated limitations. This stage actively involves both the patient and their family in addressing the chronic condition and encourages the adoption of appropriate health behaviors.

During the sensitization stage, participants in the intervention group will attend weekly sessions for four consecutive weeks (a total of four sessions), with each session lasting approximately 30 to 45 min. These sessions will take place in a quiet room with appropriate lighting and temperature, free from environmental distractions, within the Parkinson’s Association. To improve the effectiveness of the training, educational slides and pre-recorded videos on PD will be used.

The key activities in this phase primarily involve care counseling, which will be conducted in group sessions. The main topics covered in these follow-up care sessions (as outlined in Table 1) include an explanation of PD and its symptoms in a way that is comprehensible to patients and their families, the acute and chronic complications of the disease and their management, the importance and implementation of a proper diet and physical activity, and the significance of adhering to prescribed medications. Together, the first and second phases constitute the initial month of the intervention program [40].

**Table 1:** Content of the sessions conducted for the intervention group

Stage	Educational behavioral objectives	Content	Teaching method	Presenter	Duration
First stage: the familiarization stage	• Learners should express their expectations regarding CCM sessions • Scheduling in-person meetings in consultation with patients and their companions • Gathering feedback from learners on the objectives of CCM sessions	• Introducing the nurse to the patient and their companion, clarifying the nurse’s expectations at different stages of the study • Identifying and addressing the expectations and concerns of the patient and their companion • Providing recommendations on the necessity of continuity in the care relationship, emphasizing minimal disruption until the designated period ends • Establishing and agreeing on schedules for in-person and phone meetings, outlining the objectives and methods of communication and educational sessions	• Lecture • Q&A (Question and Answer) • Group Discussion	Researcher	30 to 45 min
Second stage: sensitization phase	• Learners should be able to describe the nature and symptoms of the disease • They should identify the primary and secondary symptoms of PD and distinguish them from other conditions • They should interpret information related to PD and explain the significance of each symptom in disease progression	• Explaining PD and its symptoms in a way that is understandable for patients and their families • Describing how the disease affects daily activities, social interactions, and the emotional well-being of patients	• Lecture • Group Discussion • Q&A • Posing questions aligned with educational objectives and allowing patients to respond	Researcher	30 to 45 min The first 10 min of the session will be allocated to an icebreaker activity
Third stage: sensitization phase	• Learners should be able to identify complications of a specific disease in a real-life scenario and propose the best management strategies • They should analyze the impact of not using prescribed medications on disease progression • Learners should be capable of designing a care plan to manage acute and chronic complications that may arise in themselves	• Acute and Chronic Complications of the Disease and Their Management • The Importance of Adhering to Prescribed Medications	• Lecture • Group Discussion • Q&A • Presentation of a Scenario by the Researcher to Patients and Receiving Their Responses	Researcher	30 to 45 min
Fourth stage: sensitization phase	• Learners should be able to propose appropriate solutions for managing symptoms and signs in three real-life scenarios	Management of PD Symptoms and Signs	• Lecture • Group Discussion • Q&A • Presentation of a Scenario by the Researcher to Patients and Receiving Their Responses	Researcher	30 to 45 min
Fifth stage: sensitization phase	• Learners should be able to design a healthy meal plan for 1 day • Learners should explain the benefits of physical activity in PD to their peers • Learners should list several simple strategies to enhance home safety • Learners should select appropriate assistive devices to facilitate walking • Learners should develop a simple plan to reduce the risk of slipping in the bathroom	• The Importance of Diet and How to Follow It • The Importance of Physical Activity and How to Maintain It • Strategies for Increasing Home Safety and Using Assistive Devices to Facilitate Walking and Maintain Balance	• Lecture • Group Discussion • Q&A	Researcher	30 to 45 min
Sixth stage: control phase	• Learners are expected to have sufficient knowledge of previous sessions and actively share their understanding during the review of past topics • Learners should express any questions or challenges they face and implement the solutions provided by the researcher • Learners should actively describe the health behaviors they have adopted since completing the sensitization sessions	• Continuation of Continuous Care Consultations to Sustain Health Behaviors Based on Patients’ Needs and Challenges • Encouraging Patients to Maintain Healthy Behaviors	• Phone Call and Q&A	Researcher	30 to 45 min
Seventh stage: control phase	• Learners are expected to have sufficient knowledge of previous sessions and actively share their understanding during the review of past topics • Learners should express any questions or challenges they face and implement the solutions provided by the researcher • Learners should actively describe the health behaviors they have adopted since completing the sensitization sessions	• Continuation of continuous care Consultations to Sustain Health Behaviors Based on Patients’ Needs and Challenges • Encouraging Patients to Maintain Healthy Behaviors	• Phone Call and Q&A	Researcher	30 to 45 min
Eighth stage: control phase	• Learners are expected to have sufficient knowledge of previous sessions and actively express their understanding during the review of past topics • Learners should articulate any questions or challenges they face and implement the solutions provided by the researcher • Learners should actively describe the health behaviors they have adopted since the completion of the sensitization sessions	• Continuation of continuous Care Consultations to Sustain Health Behaviors Based on Patients’ Needs and Challenges • Encouraging Patients to Maintain Healthy Behaviors	• Phone Call and Q&A	Researcher	30 to 45 min
Ninth stage: control phase	• Learners are expected to have sufficient knowledge of previous sessions and actively express their understanding during the review of past topics • Learners should articulate any questions or challenges they face and implement the solutions provided by the researcher • Learners should actively describe the health behaviors they have adopted since the completion of the sensitization sessions	• Continuation of continuous Care Consultations to Sustain Health Behaviors Based on Patients’ Needs and Challenges • Encouraging Patients to Maintain Healthy Behaviors	• Phone Call and Q&A	Researcher	30 to 45 min
Tenth stage: evaluation phase	• Completion of Questionnaires by Participants • Learners should express any new or unresolved issues and listen to the solutions provided by the researcher or the experiences of other participants regarding the existing problem	• Review of the Care Process, Evaluation of Key Indicators through Recompletion of Questionnaires, and Monitoring and Controlling Patient Behavior and the Impact of Provided Training • Assessment of Unresolved Issues or the Emergence of New Problems	• Lecture • Q&A • Group Discussion	Researcher	30 to 45 min

In the monitoring phase, follow-up care counseling continues to reinforce the maintenance of healthy behaviors. This phase is carried out over 4 weeks through four telephone calls (one call per week) with the patient and, if necessary, with their caregiver. The content of these calls is tailored to the patient's specific problems and needs.

A key component of this phase is encouraging the patient to sustain healthy behaviors and referring them to a specialist if required. Additionally, a contact number will be provided to patients and their caregivers, allowing them to reach out with any questions and receive necessary guidance [40].

The evaluation phase, which is the final step of the intervention, involves assessing the care process, evaluating key indicators through the reassessment of questionnaires, and monitoring the patient’s behavior to determine the impact of the provided education. Additionally, any unresolved issues or newly emerging problems will be examined [40].

### Criteria for discontinuing or modifying allocated interventions {11b}

Participants have the right to withdraw from the study at any time and for any reason, without facing any negative consequences. They will be assured that their decision to discontinue participation will not affect the quality of their clinical care in any way.

Additionally, participants may request to stop the intervention at any stage of the study. If a participant experiences emotional distress, health-related complications, or any other condition that makes continuation inappropriate, the intervention may be paused or terminated based on the researcher’s clinical judgment. All reasons for discontinuation or modification will be recorded and documented accordingly.

### Strategies to improve adherence to interventions {11c}

To improve adherence to the intervention protocol, participants will receive weekly reminders via text messages to attend the scheduled sessions. Additionally, regular follow-up calls will be made by the researcher to assess participant progress and address any concerns. Adherence will also be monitored by tracking attendance at intervention sessions and any required reports or questionnaires. Any missed sessions will be rescheduled, and participants will be encouraged to communicate any barriers to adherence.

*Relevant concomitant care permitted or prohibited during the trial {11d* **}.**

During the study period, participants are prohibited from participating in additional structured educational sessions related to Parkinson’s disease (e.g., nutrition or lifestyle workshops offered by organizations such as the Isfahan Parkinson’s Charity Association) to prevent confounding effects on the study outcomes. This restriction does not apply to routine medical care, including prescribed medications, physiotherapy, or other standard treatments for Parkinson’s disease, which participants are permitted to continue under the supervision of their healthcare providers to ensure their welfare is maintained.

### Provisions for post-trial care {30}

As the intervention is educational and does not involve any pharmaceutical treatments or interventions with side effects, there are no specific post-trial care requirements. However, participants will be followed up for a period of 3 months after the trial to assess any long-term impact of the intervention on their knowledge and self-management of Parkinson’s disease. If any participant experiences any issues related to the study, they will be referred to appropriate healthcare professionals for further support.

Since the intervention poses no direct risk, no compensation is anticipated for harm from trial participation. However, any concerns raised by participants during the post-trial follow-up will be addressed promptly.

### Outcomes {12}

The primary outcome is quality of life, measured using the PDQ-8, at 1 month post-intervention to assess sustained effects. Secondary outcomes include fatigue (PFS-16) and self-efficacy (GSE-17), assessed at baseline, immediately post-intervention, and 1 month post-intervention. Data collection will be coordinated via telephone, and participants will be asked to visit the Isfahan Parkinson’s Charity Association to complete the questionnaires. If a participant is unable to attend, the researcher will complete the questionnaire at the participant’s residence.

### Participant timeline {13}

#### Sample size {14}

To determine the sample size, assuming a normal distribution of quality-of-life data, the following formula will be used.

$$n= \frac{2 \times {\sigma }^{2}{ \times ({Z}_{1-\beta }+ {Z}_{1-\alpha /2})}^{2}}{{({\mu }_{1}-{\mu }_{2} )}^{2}}$$

The standard deviation in the study by Moghtaderi et al. was reported to be approximately 6 [41]. Therefore, with a power of 80% (1 − β = 80%) and assuming a confidence level of *α* = 0.05, the required sample size for each group to detect a difference of 4 units (μ₁ − μ₂ = 4) is:

$$n=\frac{2 \times {6}^{2} \times {(0.84+ 1.96)}^{2}}{{(4)}^{2}}=35.28\approx 36$$

The required sample size for each group will be 36 participants, resulting in a total of 72 samples. Considering a 10% dropout rate, a total of 80 participants will be required for the study.

### Recruitment {15} (Fig. 1)

A total of 80 patients diagnosed with PD who meet the inclusion criteria will be recruited from the Isfahan Parkinson Charity Association. After obtaining official approval from the School of Nursing and Midwifery, Isfahan University of Medical Sciences, and presenting it to the association’s authorities, the researcher will enter the study environment and screen participants based on inclusion criteria with confirmation from a neurologist specializing in movement disorders. The estimated duration for the recruitment process is approximately 2 months.

**Fig. 1:**
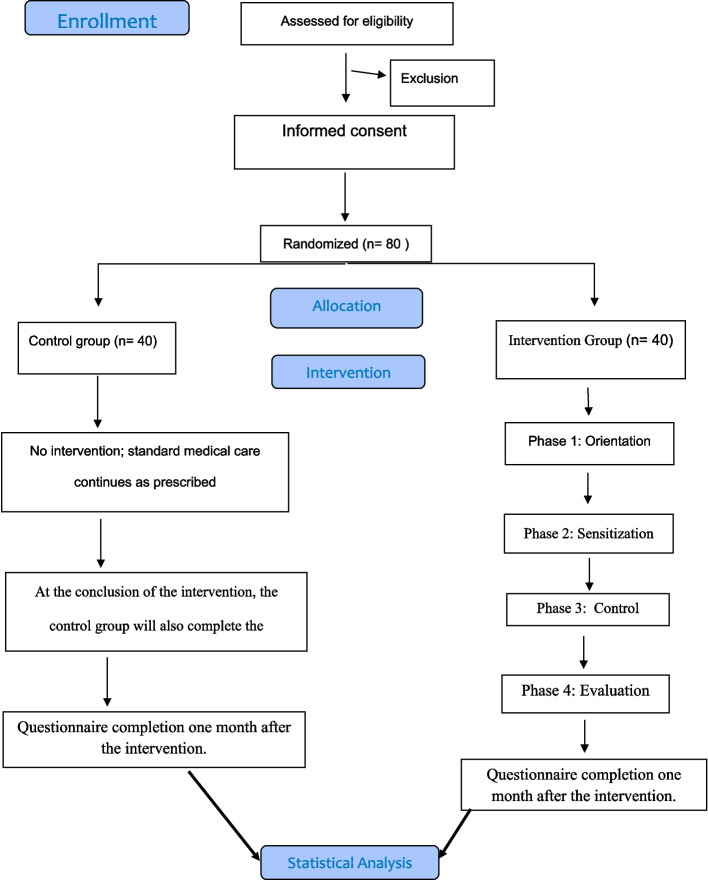
Consort Diagram

## Assignment of interventions: allocation

### Sequence generation {16a}

From all registered patients, 80 eligible participants will be selected using a convenience sampling method. They will be randomly assigned into two groups: 40 in the intervention group and 40 in the control group.

Participants will be allocated using block randomization. The intervention group will be labeled “A” and the control group “B.” The six possible block arrangements (AABB, ABBA, ABAB, BAAB, BABA, BBAA) will be written on separate cards.

A number between 1 and 6 will be randomly selected (using a random number table). For instance, if block 2 (ABBA) is chosen, the first patient will be assigned to the intervention group, the next two to the control group, and the fourth to the intervention group. This process will continue until the required sample size is reached.

### Concealment mechanism {16b}

The allocation sequence will be concealed until the interventions are assigned. Block randomization will be used to assign participants to either the intervention group (Group A) or the control group (Group B).

To prevent any bias, the block sequences will be written on separate cards or in sealed envelopes. These envelopes will be kept secure and will not be accessible to the researchers who are enrolling participants. This ensures that the allocation sequence remains concealed until the moment of random assignment.

### Implementation {16c}

The random allocation sequence will be generated using a random number table.

Mahshid Ebrahimiyan Tadi, the principal investigator, will be responsible for enrolling participants, obtaining informed consent, and assigning them to the intervention or control groups based on the pre-generated block randomization sequence.

## Assignment of interventions: blinding

### Who will be blinded {17a}

Due to the nature of the intervention, this study will not be blinded. The researcher administering the intervention and the patients will be aware of their group assignments, as participation in the intervention requires awareness of the assigned group. Therefore, concealment of allocation is not feasible, and the study is designed as an open-label trial.

### Procedure for unblinding if needed {17b}

Blinding is not applicable in this trial; therefore, unblinding procedures are not required.

## Data collection and management

### Plans for assessment and collection of outcomes {18a}

In this study, data will be collected using a four-part questionnaire, as described below:

- Demographic and Clinical Information Questionnaire: This section gathers information on variables such as age, gender, educational level, occupation, marital status, income level, support resources, history of physical and mental illnesses, duration of PD, medications used, and family history of PD.
- Parkinson’s Disease Fatigue Scale Questionnaire: In this study, the PFS-16 will be used to assess fatigue levels in patients with PD. This questionnaire was developed in 2005 by Brown et al. in the UK to evaluate the impact of fatigue on daily life activities in these patients [42]. The PFS-16 consists of 16 items**,** with 7 questions assessing the experience and physical impact of fatigue**,** while the remaining 9 questions evaluate the effect of fatigue on daily activities and performance**.** Participants are required to respond based on their fatigue experiences over the past 2 weeks. Responses are rated on a Likert scale with the following options: strongly disagree [1], disagree [2], neutral [3], agree [4], and strongly agree [5]. A total score of 8 or higher indicates the presence of significant fatigue.
- General Self-Efficacy Questionnaire: In this study, the GSE-17 will be used to assess self-efficacy levels in patients with PD. This questionnaire was developed in 1982 by Sherer et al. to determine various levels of general self-efficacy. Sherer and Maddux suggested that, regardless of the individual items, this scale consists of three main factors**:** the willingness to initiate behavior, the willingness to extend effort to complete a task, and perseverance in overcoming obstacles [43]. The scale consists of 17 items, rated on a 5-point Likert scale from 1 to 5. Specifically, in items 1, 3, 8, 9, 13, and 15, a response of “Strongly Agree” is scored as 5, while “Strongly Disagree” is scored as 1**.** In the remaining items, the scoring is reversed. The maximum possible score is 85, while the minimum is 17. A higher score indicates a greater sense of self-efficacy. Scores ranging from 17 to 34 indicate low self-efficacy, 34 to 51 indicate moderate self-efficacy, and 51 to 85 indicate high self-efficacy**.**
- Short Form Parkinson’s Disease Quality of Life Questionnaire: In this study, the PDQ-8 will be used to assess the QOL in patients with PD. This questionnaire was developed in 1997 by Jenkinson et al. with the aim of providing a shorter and more efficient method for measuring the QOL in PD patients, thereby improving ease of use in clinical settings. The PDQ-8 is a shortened version of the 39-item Parkinson’s Disease Questionnaire (PDQ-39). The PDQ-39 consists of 8 domains, and the PDQ-8 is derived by selecting one question from each domain of the PDQ-39. These eight questions were chosen based on their ability to reflect the most important aspects of QOL related to PD [44]. This questionnaire consists of 8 questions, each with response options ranging from never (score 0) to always (score 4), using a Likert scale. The total score is calculated by summing the scores of each question. A higher score indicates poorer QOL.

### Plans to promote participant retention and complete follow-up {18b}

Participants will receive weekly text reminders and follow-up calls to support adherence. Attendance and completion of required tasks will be monitored, and missed sessions will be rescheduled when possible.

Participants who discontinue the intervention or deviate from the protocol will not be followed for outcome data, unless they voluntarily request to receive the study results or the educational care materials. In such cases, these materials will be provided, but no further data will be collected for analysis.

### Data management {19}

Data will be collected using paper-based questionnaires completed by participants. Responses will be entered into a password-protected laptop by the researcher. Each participant will be assigned a unique code, and no names or personal identifiers will be stored. As the data consist of numerical values only, no advanced security measures are applied. Data quality will be ensured through careful data entry and periodic checks for completeness and consistency.

### Confidentiality {27}

Personal information about participants will be collected using paper-based forms and assigned unique codes to ensure anonymity. Data will be stored securely on a password-protected laptop, and only the research team will have access to the information. Participant names and identifying details will not be recorded or shared with any external parties. After the study, data will be securely stored for future reference or analysis, or destroyed, depending on the study’s requirements. All efforts will be made to protect participant confidentiality before, during, and after the trial.

*Plans for collection, laboratory evaluation*,* and storage of biological specimens for genetic or molecular analysis in this trial/future use {33}.*

No biological specimens will be collected for genetic or molecular analysis in this trial. As a result, there are no plans for the collection, laboratory evaluation, or storage of such specimens.

## Statistical methods

### Statistical methods for primary and secondary outcomes {20a}

For data analysis, descriptive statistics, including mean and standard deviation, will first be calculated. In inferential statistics, to compare changes in the three quantitative variables across three time points (before the intervention, immediately after the intervention, and 1 month later) between the control and intervention groups, repeated measures analysis of covariance (ANCOVA) will be used, controlling for covariates. The Kolmogorov–Smirnov test will be applied to assess data normality, while Levene’s test will be used to examine variance homogeneity. All analyses will be performed using SPSS software, version 25.

### Interim analyses {21b}

There will be no interim analyses conducted for this educational and care-based study, as it is considered low-risk. Since there are no safety concerns or critical endpoints that would require early stopping, the study will proceed according to the established protocol. Therefore, no stopping guidelines are applicable.

### Methods for additional analyses (e.g., subgroup analyses) {20b}

This study includes both an intervention group and a control group. The primary and secondary outcomes will be analyzed by comparing these two groups using appropriate statistical methods. Subgroup analyses may be conducted based on characteristics such as age, gender, or baseline health status to explore any differential effects of the intervention. Adjusted analyses will be performed to control for potential confounding variables.

### Methods in analysis to handle protocol non-adherence and any statistical methods to handle missing data {20c}

Participants who fail to attend more than two sessions, do not actively cooperate, or do not complete the required questionnaires will be considered non-adherent to the protocol. Initially, they will be reminded to complete the questionnaires or attend the sessions. If they continue to not comply, they will be excluded from the analysis. For missing data, any participants with incomplete data will be excluded from the final analysis.

### Plans to give access to the full protocol, participant-level data, and statistical code {31c}

The full protocol, participant-level data, and statistical code will not be made publicly available during the course of the study. However, after the study is completed and the results are published in a peer-reviewed journal, these materials will be available upon request. This ensures transparency and reproducibility while maintaining participant confidentiality. Access will be granted to authorized researchers or institutions, with any personally identifiable information removed to protect privacy.

## Oversight and monitoring

### Composition of the coordinating centre and trial steering committee {5d}

The trial will be overseen by the supervising professor and the advisory committee, who will monitor the progress of the study and ensure that all aspects of the study are conducted according to the protocol. The supervising professor is responsible for providing high-level guidance, while the advisory committee will offer additional support and advice on specific areas of the study.

Any issues or concerns arising during the trial will be addressed by the supervising professor and advisory committee, who will make decisions regarding the study’s progress. Ethical approval for the study has been granted by the university’s ethics committee, ensuring the study meets all ethical standards.

### Composition of the data monitoring committee, its role and reporting structure {21a}

This study does not have a Data Monitoring Committee (DMC) due to its educational and low-risk nature. The study is not expected to involve significant risks or adverse events that would require ongoing oversight by an independent committee.

However, the progress of the study and the integrity of the data will be monitored by the supervising professor and the advisory committee, who will ensure that the study follows the protocol and ethical guidelines. Any concerns or issues arising during the study will be addressed by these individuals, who will make decisions about the continuation of the study.

### Adverse event reporting and harms {22}

In this study, participants will be closely monitored for any adverse events or unintended effects resulting from the intervention. Participants will be encouraged to report any negative effects they experience during the trial, and routine assessments will be conducted at each follow-up session to identify any potential adverse events.

All reported adverse events, whether solicited or spontaneously reported by participants, will be documented and assessed for severity. If an adverse event occurs, the research team will evaluate whether it is related to the intervention and determine the necessary course of action, including whether the participant should continue in the study or whether the intervention needs to be modified.

Adverse events will be reported to the university’s ethics committee and any relevant regulatory bodies, as required. The research team will take appropriate steps to manage and address any adverse effects, ensuring the safety and well-being of all participants.

### Frequency and plans for auditing trial conduct {23}

In this study, the oversight and monitoring of the trial will be carried out by the supervising professor and advisory committee. They will continuously review the progress of the study and ensure that it is conducted according to the protocol and ethical guidelines. Additionally, the university’s ethics committee may perform random oversight or additional monitoring if necessary.

### Plans for communicating important protocol amendments to relevant parties (e.g., trial participants, ethical committees) {25}

In accordance with the SPIRIT guidelines, the following plans outline how important protocol amendments will be communicated to relevant parties involved in the trial, such as trial participants, ethical committees (REC/IRB), investigators, trial registries, journals, and regulators.

- Trial Participants: If there are any amendments that impact the eligibility criteria, outcomes, or the overall intervention, trial participants will be informed promptly and clearly. Communication will be through written notifications, and if necessary, verbal communication will be arranged by the study team. Participants will also be provided with updated consent forms if the protocol changes affect their participation.
- Ethical Committees (REC/IRB): All significant protocol modifications, particularly those that may affect participant safety, eligibility, or the scientific integrity of the study, will be submitted to the relevant Ethics Committees (REC/IRB) for review and approval. Any changes that are deemed necessary by the REC/IRB will be addressed before implementation.
- Investigators and Trial Staff: All investigators and trial staff will be immediately informed of the amendments through official correspondence. Training sessions or briefings will be organized if necessary to ensure that everyone involved is fully aware of the changes and their implications for trial conduct.
- Trial Registries: The protocol amendments will be updated in the relevant trial registries to ensure that public records of the trial reflect the most accurate and up-to-date information.
- Journals and Publications: If the trial has already been published or if there are plans for publication, any relevant amendments will be communicated to the journals in order to update previously submitted or published protocols and results.
- Regulators: If the protocol amendments are substantial, especially in terms of safety or scientific aspects, the relevant regulatory bodies (e.g., FDA, EMA) will be notified in accordance with regulatory requirements. The necessary documentation will be submitted to ensure compliance with regulatory standards.

By following these communication plans, we aim to ensure transparency, maintain the integrity of the trial, and ensure that all parties are kept informed of the protocol amendments in a timely and efficient manner.

### Dissemination plans {31a}

The results of this clinical trial will be submitted for publication in reputable peer-reviewed scientific journals. In addition, a summary of the findings will be shared with trial participants in a clear and understandable format. Healthcare professionals and other relevant stakeholders will be informed through professional networks and scientific communications. Primary outcome data and other relevant datasets will be made publicly available through trial result registries or open-access repositories, where applicable. There are no publication restrictions imposed by the sponsor or any other party.

## Discussion

Based on previous studies, various methods have been implemented to manage fatigue, enhance self-efficacy, and improve the QOL in patients with PD. However, each approach has faced challenges, one of which is the lack of a comprehensive strategy to address these variables effectively.

The Ahmadi CCM primarily focuses on caring for individuals with chronic diseases who require ongoing supervision and support. One of the core principles of this model is patient and family education and empowerment. Patients learn how to manage their condition and actively participate in their own treatment and self-care. The educational components of this model cover medication management, lifestyle modifications, and healthy nutrition.

Another key principle of this model is supporting patient self-care. Patients are encouraged to take greater responsibility for their health and well-being. Additionally, continuous monitoring and follow-up play a crucial role in the Ahmadi model. Regular follow-up sessions and ongoing communication—either in person or via telephone—are conducted to assess the patient’s health status.

Overall, this model provides a comprehensive and proactive approach to chronic disease management, placing the patient at the center of the care process and enabling them to play an active role in maintaining and improving their health.

## Trial status

- Protocol version number and date: Protocol, February 6, 2025.
- Date recruitment began: Recruitment is expected to begin around June 15, 2025.
- Estimated recruitment completion date: Recruitment is expected to be completed by July 15, 2025

## Supplementary Information

Supplementary Material 1

## Data Availability

The results of this clinical trial will be submitted for publication in reputable peer-reviewed scientific journals. Additionally, some of the data, including key information regarding the primary outcome, will be made publicly available.

## Abbreviations

PD: Parkinson’s disease
QOL: Quality of life
CCM: Continuous care model
IRCT: Iranian Registry of Clinical Trials
DALYs: Disability-adjusted life years
WHO: World Health Organization
HRQOL: Health-related quality of life
PFS-16: Parkinson’s Disease Fatigue Scale 16
GSE-17: General Self-Efficacy Scale
PDQ-8: Parkinson’s Disease Questionnaire-8
SPIRIT checklist: Standard Protocol Items: Recommendations for Interventional Trials
Q&A: Question and Answer

## References

[1] Safiri S, Noori M, Nejadghaderi SA, Mousavi SE, Sullman MJ, Araj-Khodaei M, et al. The burden of Parkinson’s disease in the Middle East and North Africa region, 1990–2019: results from the global burden of disease study 2019. BMC Public Health. 2023;23(1): 107. 36642724 10.1186/s12889-023-15018-x PMC9841703

[2] Dorsey ER, Elbaz A, Nichols E, Abbasi N, Abd-Allah F, Abdelalim A, et al. Global, regional, and national burden of Parkinson’s disease, 1990–2016: a systematic analysis for the Global burden of disease study 2016. Lancet Neurol. 2018;17(11):939–53. 30287051 10.1016/S1474-4422(18)30295-3 PMC6191528

[3] WHO. Parkinson disease 2023 [Available from: https://www.who.int/news-room/fact-sheets/detail/parkinson-disease.

[4] Hosseinzadeh A, Baneshi MR, Sedighi B, Kermanchi J, Haghdoost AA. Estimation of Parkinson’s disease prevalence and its geographical variation in Iran. J Mazandaran Univ Med Sci. 2021;31(200):113–24.

[5] Rocca WA. The burden of Parkinson’s disease: a worldwide perspective. Lancet Neurol. 2018;17(11):928–9. 30287052 10.1016/S1474-4422(18)30355-7

[6] Whetten-Goldstein K, Sloan F, Kulas E, Cutson T, Schenkman M. The burden of Parkinson’s disease on society, family, and the individual. J Am Geriatr Soc. 1997;45(7):844–9. 9215336 10.1111/j.1532-5415.1997.tb01512.x

[7] Sjödahl Hammarlund C, Westergren A, Åström I, Edberg A-K, Hagell P. The impact of living with Parkinson’s disease: balancing within a web of needs and demands. Parkinson’s Disease. 2018;2018(1):4598651. 30151098 10.1155/2018/4598651 PMC6087577

[8] Pfeiffer RF. Non-motor symptoms in Parkinson’s disease. Parkinsonism Relat Disord. 2016;22:S119–22. 26372623 10.1016/j.parkreldis.2015.09.004

[9] Siciliano M, Trojano L, Santangelo G, De Micco R, Tedeschi G, Tessitore A. Fatigue in Parkinson’s disease: a systematic review and meta-analysis. Mov Disord. 2018;33(11):1712–23. 30264539 10.1002/mds.27461

[10] Stocchi F, Abbruzzese G, Ceravolo R, Cortelli P, D’Amelio M, De Pandis MF, et al. Prevalence of fatigue in Parkinson disease and its clinical correlates. Neurology. 2014;83(3):215–20. 24928125 10.1212/WNL.0000000000000587

[11] Lin I, Edison B, Mantri S, Daeschler M, Kopil K, Marras C, Chahine L. Triggers and Alleviating Factors for Fatigue in Parkinson’s Disease: 736. Mov Disord. 2020;35:S330. 10.1371/journal.pone.0245285 PMC7861907 33540422

[12] Nassif DV, Pereira JS. Fatigue in Parkinson’s disease: concepts and clinical approach. Psychogeriatrics. 2018;18(2):143–50. 29409156 10.1111/psyg.12302

[13] Estrada-Bellmann I, Meléndez-Flores JD, Cámara-Lemarroy CR, Castillo-Torres SA. Determinants of self-efficacy in patients with Parkinson’s disease. Arq Neuropsiquiatr. 2021;79(8):686–91. 34550188 10.1590/0004-282X-ANP-2020-0185

[14] Prewitt CM, Charpentier JC, Brosky JA, Urbscheit NL. Effects of dance classes on cognition, depression, and self-efficacy in Parkinson’s disease. Am J Dance Ther. 2017;39:126–41.

[15] Lau SC, Bhattacharjya S, Fong MW, Nicol GE, Lenze EJ, Baum C, et al. Effectiveness of theory-based digital self-management interventions for improving depression, anxiety, fatigue and self-efficacy in people with neurological disorders: a systematic review and meta-analysis. J Telemed Telecare. 2022;28(8):547–58. 32954920 10.1177/1357633X20955122 PMC8145956

[16] Pant N. Spirituality. Mental Health and Quality of Life: Springer; 2023.

[17] Zhao N, Yang Y, Zhang L, Zhang Q, Balbuena L, Ungvari GS, et al. Quality of life in Parkinson’s disease: a systematic review and meta-analysis of comparative studies. CNS Neurosci Ther. 2021;27(3):270–9. 33372386 10.1111/cns.13549 PMC7871788

[18] Gui Y, Zhou Y. High-quality nursing intervention can improve negative emotions, quality of life and activity of daily living of elderly patients with Parkinson’s disease. Am J Transl Res. 2021;13(5):4749. 34150055 PMC8205788

[19] Schrag A, Quinn N. What contributes to quality of life in Parkinson’s disease: a re-evaluation. J Neurol Neurosurg Psychiatry. 2020;91(6):563–5. 32139651 10.1136/jnnp-2019-322379

[20] Pablo Martínez-Martin FS, Heinz Reichmann. Quality of Life in Parkinson’s Disease – Patient, Clinical and Research Perspectives 2014 [Available from: https://touchneurology.com/movement-disorders/journal-articles/quality-of-life-in-parkinsons-disease-patient-clinical-and-research-perspectives/.

[21] Rahimi Gharibvand M, Nasr Isfahani M. Families about continuous care at hospitals affiliated to Isfahan University of Medical Sciences in 2014. J Diabetes Nurs. 2014;2(3):38–48.

[22] Ahmadi F, Ghofranipour F, Abedi H, Arefi S, Faghihzadeh S. The design of continous care model for the control of coronary artery disease. 2002.

[23] Jalalmarvi F. Assessing the impact of continuous care model on the treatment of chronic diseases. Paramedical Sciences and Military Health. 2018;13(2):35–43.

[24] Moosavinasab SMM, Vahedian-Azimi A, Salesi M, Vahedi E, Zarchi A, KhoshFetrat M, Bashar F. A review of 17 years of application of a continuous care model on the consequences of acute and chronic diseases: describing and assessing the quality of methodology of papers. J Mil Med. 2018;20(1):27–55.

[25] Sadeghi SM. Effect of applying continuous care model on quality of life in heart failure patients. Int J Behav Sci. 2009;3(1):9–13.

[26] AKBARI O, VAGHAR SSA, SAADATJOO SA, KAZEMI T. Effect of continuous care model on the self-efficacy of patients with myocardial infarction in controlling disease complications. 2015.

[27] Rahimi A, Ahmadi F, Gholyaf M. Effects of applying continuous care model on quality of life in hemodialysis patients. 2006.

[28] Tayebi A, Rahimi A, Einollahi B, Mirsadeghi A, Hashemi S. The effect of continues care model on adherence to treatment in hemodialysis patients. J Crit Care Nurs. 2019;12(2):42–7.

[29] Pourshaban Z, Moghimian M, Jouzi M. The effect of continuous care model on the burden care of family caregivers of stroke patients. J Nurs Educ. 2023;12(1):27–35.

[30] Jalal MF, KORDI M, MAZLOM SR, REZAEI TF. Comparing the effect of training based on continuous care model and telehealth on severity of insomnia in pregnant women. 2019.

[31] Moradi M, Ebrahimi H, Goli S, Khajeh M. The effect of sleep health education program based on continuous care model on sleep quality in the elderly. Iran J Health Educ Health Promot. 2022;10(2):171–84.

[32] Khodaveisi M, Ashtarani F, Mohammadi N, Mahjub H, Mazdeh M. The effect of continuous care on quality of life in multiple sclerosis patients. Avicenna J Nurs Midwifery Care. 2014;22(2):64–73. 10.4103/1735-9066.208170 PMC5494953 28706548

[33] HOJAT M, KARIMYAR JM, KARAMI Z. Effect of continuous care model on sleep quality and dialysis adequacy of hemodialysis Patients: a clinical trial study. 2015.

[34] Khankeh H, Anjomanian V, AHMADI FE, FALAHI KM, RAHGOZAR M, RANJBAR M. Evaluating the effectiveness of continuous care model on quality of life in discharged schizophrenic patients from Sina educational and medical center, Hamedan, 2007. 2010.

[35] Hakim A, Valavi E, MADHOOSHI MS, Latifi S, Dashtbozorgi B. THE EFFECT OF CONTINUOUS CARE MODEL ON PARENTS’KNOWLEDGE AND CONTROLLING SYMPTOMS AND RECURRENCE IN CHILDREN WITH NEPHROTIC SYNDROME. 2016.

[36] Isernia S, Di Tella S, Pagliari C, Jonsdottir J, Castiglioni C, Gindri P, et al. Effects of an innovative telerehabilitation intervention for people with Parkinson’s disease on quality of life, motor, and non-motor abilities. Front Neurol. 2020;11: 846. 32903506 10.3389/fneur.2020.00846 PMC7438538

[37] Jethani PM, Toglia J, Foster ER. Cognitive self-efficacy in Parkinson’s disease. OTJR Occup Ther J Res. 2024;44(4):625–31. 10.1177/15394492231206346 PMC11408982 37905522

[38] Chen Y, Lu T, Jiang X, Huang X. The effectiveness of specialized nursing interventions for patients with Parkinson disease: a randomized controlled study protocol. Medicine (Baltimore). 2021;100(2): e23972. 33466136 10.1097/MD.0000000000023972 PMC7808514

[39] Moghtaderi M, Saffarinia M, Zare H, Alipour A. Effectiveness of the package of hope therapy based on positivist approach on the self-efficacy and loneliness of Parkinson patients. J Health Psychol. 2020;8(32):73–92.

[40] Asheri S, Nasrollah S, Nasrabadi T. The effect of continuous care model on self-care in patients with colon cancer. J Nurs Educ. 2022;11(5):68–79.

[41] Moghtaderi M, Saffarinia M, Zare H, Alipour A, Chitsaz A. Effectiveness of the mindfulness therapy on the life quality and psychological wellbeing of Parkinson patients. Journal of Psychological Science. 2019;18(83):2213–21.

[42] Brown RG, Dittner A, Findley L, Wessely SC. The Parkinson fatigue scale. Parkinsonism Relat Disord. 2005;11(1):49–55. 15619463 10.1016/j.parkreldis.2004.07.007

[43] Sherer M, Maddux JE, Mercandante B, Prentice-Dunn S, Jacobs B, Rogers RW. The self-efficacy scale: construction and validation. Psychol Rep. 1982;51(2):663–71.

[44] Jenkinson C, Ray F, Viv P, Richard G, Hyman N. The PDQ-8: development and validation of a short-form parkinson’s disease questionnaire. Psychol Health. 1997;12(6):805–14.

