# Supplementary material for: Effect of a continuous care model-based program on fatigue, self-efficacy, and quality of life in patients with Parkinson’s disease: study protocol for a randomized controlled trial
Source: Trials. 2025 Sep 25;26:355. doi: 10.1186/s13063-025-09057-5 (PMC12465541; doi:10.1186/s13063-025-09057-5)
Supplement: Supplementary file 1 — Supplementary Material 1 [file 13063_2025_9057_MOESM1_ESM.docx]

**Informed Consent for Participation in a Research Project / Thesis**

**Title of the Study:**
*The Effect of a Program Based on the Continuous Care Model on Fatigue, Self-Efficacy, and Quality of Life in Patients with Parkinson’s Disease Referred to the Parkinson’s Association of Isfahan Province in 2025*

**Principal Investigators:**
Dr. Shahla Abolhasani and Mahshid Ebrahimiyan Tadi

**Affiliated Institution:**
Isfahan University of Medical Sciences, School of Nursing and Midwifery

**Dear Sir/Madam,**

You (or your child / the individual under your care), named: ___________________________
National ID (optional): ___________________________

are invited to participate in the above-mentioned research project. The relevant information about this study is provided in this document. Please note that participation in this study is entirely voluntary, and you are free to choose whether or not to take part.

You are not required to make an immediate decision. You may take up to 48 hours to consider your participation, ask any questions you may have from the research team, and consult with anyone you wish before making a decision.
Before signing this consent form, please ensure that you fully understand the information provided and that all your questions have been adequately answered.

1. **I understand that the objectives of this study are as follows:**
   To assess the effect of a program based on the Continuous Care Model on fatigue, self-efficacy, and quality of life in patients with Parkinson’s disease who are referred to the Parkinson’s Association of Isfahan Province in 2025.
2. **I understand that the procedures of this study and the participation of myself / my child / the individual under my care are as follows:**

**a.** I understand that participants will be randomly assigned to either the intervention group or the control group. Both groups will take part in this study.

**b.** If assigned to the intervention group, the program will be conducted over a period of two months at the Parkinson’s Association.

I understand that my / my child’s / the individual under my care’s participation in this study is completely voluntary, and I/we are under no obligation to take part. I have been assured that if I / my child / the individual under my care choose(s) not to participate, we will not be denied access to standard diagnostic or therapeutic care. Our treatment relationship with the healthcare provider or center will not be affected in any way.

1. **I understand that even after agreeing to participate, I / my child / the individual under my care may withdraw from the study at any time, simply by notifying the investigator. Such withdrawal will not affect access to standard medical care for myself / my child / the individual under my care.**
2. **I understand that if any physical or psychological complications arise during or after the course of the study as a result of participation, the researcher will be responsible for covering all treatment-related expenses and for providing appropriate compensation.**
3. **I understand that, in my / my child’s / the individual under my care’s current condition, alternative approaches such as the routine care and educational services provided by the Parkinson’s Association may be used instead of the proposed intervention in this study.**
4. **I have been assured that, should there be any changes in the implementation of the study or if new information becomes available during the study that may influence my decision to continue participation, I will be informed accordingly. The university’s ethics committee will also be notified, and I will be asked to review and re-sign the informed consent form if necessary.**
5. **I understand that all individuals involved in this research are obligated to keep all information related to me / my child / the individual under my care strictly confidential. Only aggregated and anonymized results may be published, without revealing any personal identifiers.**
6. **I understand that the Research Ethics Committee, whose role is to ensure the protection of my rights, may have access to my / my child’s / the individual under my care’s information as part of its oversight responsibilities.**
7. **I understand that there will be no cost to me / my child / the individual under my care for the interventions or procedures carried out as part of this study.**
8. **Ms. Mahshid Ebrahimiyan Tadi has been introduced to me as the designated contact person for this study. I have been informed that I may reach out to her at any time should I have any questions or concerns regarding participation in the study, and she will provide guidance and support.**

Her contact details have been provided to me as follows:
• **Work Address:** School of Nursing and Midwifery, Isfahan University of Medical Sciences
• **Work Phone:** [--]
• **Mobile Phone:** +98 903 454 5271

1. **I understand that if I have any concerns or complaints regarding the research process or the conduct of the research team, I may contact the Research Ethics Committee of Isfahan University of Medical Sciences, either verbally or in writing.**
   **Address:** Hezar Jarib Street, Building No. 4, 2nd Floor, Secretariat of the Research Ethics Committee, Isfahan University of Medical Sciences
   **Phone:** +98 31 37923054
2. **I understand that this informed consent form has been prepared in two copies. One signed copy will be given to me, and the other will be kept by the principal investigator.**

**I hereby confirm that I have read and understood all of the above information. Based on this, I give my informed consent for myself / my child / the individual under my care, as identified above, to participate in this research study.**

**Signature of Researcher**            **Signature of Participant / Legal Guardian**

Participant’s Mobile Number: ......................................
